# Uncovering the mechanism of Kang-ai injection for treating intrahepatic cholangiocarcinoma based on network pharmacology, molecular docking, and *in vitro* validation

**DOI:** 10.3389/fphar.2023.1129709

**Published:** 2023-03-02

**Authors:** Fei Song, Chang-Liang Lu, Cheng-Gui Wang, Chen-Wei Hu, Yu Zhang, Tian-Lun Wang, Lu Han, Zhong Chen

**Affiliations:** ^1^ Department of Hepatobiliary Surgery, Affiliated Hospital of Nantong University, Medical School of Nantong University, Nantong, China; ^2^ Jiangsu Vocational College of Medicine, Yancheng, China

**Keywords:** Kang-ai injection, intrahepatic cholangiocarcinoma, network pharmacology, molecular docking, PI3K/Akt signaling pathway

## Abstract

**Objective:** Kang-ai injection (KAI) has been a popular adjuvant treatment for solid tumors, but its anti-tumor mechanism in intrahepatic cholangiocarcinoma (ICC) remains poorly understood. This study applied a network pharmacology-based approach to unveil KAI’s anti-tumor activity, key targets, and potential pharmacological mechanism in ICC by integrating molecular docking and *in vitro* validation.

**Methods:** The KAI-compound-target-ICC network was constructed to depict the connections between active KAI compounds and ICC-related targets based on the available data sources. The crucial ingredients, potential targets, and signaling pathways were screened using GO, KEGG enrichment analysis, and the PPI network. Molecular docking was performed to visualize the interactions between hub targets and components. *In vitro* experiments were carried out to validate the findings.

**Results:** Among the 87 active components of KAI and 80 KAI-ICC-related targets, bioinformatics analysis identified quercetin as a possible candidate. GO and KEGG enrichment analysis indicated that the PI3K-AKT signaling pathway might be essential in ICC pharmacotherapy. The PPI network and its sub-networks screened 10 core target genes, including AKT1 and IL1β. Molecular docking results showed stable binding between AKT1 and IL1β with KAI active ingredients. The *in vitro* experiments confirmed that KAI might suppress the proliferation of ICC cell lines by inhibiting the PI3K/AKT signaling pathway, consistent with the network pharmacology approach and molecular docking predictions.

**Conclusion:** The study sheds light on KAI’s biological activity, potential targets, and molecular mechanisms in treating ICC and provides a promising strategy for understanding the scientific basis and therapeutic mechanisms of herbal treatments for ICC. This research has important implications for developing new, targeted therapies for ICC and highlights the importance of network pharmacology-based approaches in investigating complex herbal formulations.

## Introduction

Intrahepatic cholangiocarcinoma (ICC) is a type of liver cancer that is second in prevalence only to hepatocellular carcinoma (HCC). It accounts for approximately 5%–10% of all primary liver cancers and has seen a rising incidence rate in recent years (Nuzzo et al., 2009; Sirica et al., 2009; Siegel et al., 2017). While surgery remains the preferred method for achieving a cure in patients with intrahepatic cholangiocarcinoma, a significant number of individuals cannot undergo this procedure at the time of diagnosis due to the advanced or inoperable nature of their disease. Timely diagnosis and systematic treatment are crucial for successful outcomes (Bridgewater et al., 2014; Kim et al., 2016). Despite undergoing surgical procedures such as local ablation or hepatic artery chemoembolization, some patients remain susceptible to postoperative recurrence (Wu et al., 2018). In recent years, targeted medications and immunotherapies have gained widespread recognition, leading to numerous clinical trials of targeted agents and immune checkpoint inhibitors (Rizzo et al., 2021; Viscardi et al., 2022). Nevertheless, as representatives of second-line ICB after sorafenib therapy failure, Nivolumab and Camrelizumab, among others, have shown unsatisfactory objective response rates (only about 15%) in recent large clinical trials (El-Khoueiry et al., 2017; Qin et al., 2020). Further investigation into potential anticancer drugs and techniques is imperative to change the current treatment paradigm for ICC.

Traditional Chinese Medicine (TCM) has been a cornerstone of health and wellness for millennia, embracing a holistic approach to promoting optimal balance, preventing ailments, and treating conditions. In particular, TCM holds great promise in managing complex conditions such as cancer tumors, immune disorders, and cardiovascular diseases (Xu et al., 2019). TCM can be considered a valuable resource and treasure trove for modern pharmaceutical development. As a hallmark of integrative oncology therapy in China, it plays a distinctive role in mitigating adverse effects, reducing the risk of recurrence, and enhancing the quality of life (Luo et al., 2021). On account of the advantages of TCM in tumor treatment, such as its multi-targeted approach and reduced drug resistance, the utilization of TCM in the treatment of intrahepatic cholangiocarcinoma has also achieved a commendable therapeutic outcome (Liu et al., 2019). However, TCM possesses the characteristic of multi-component and multi-targeted action, resulting in complex interactions among these targets, leading to an unclear molecular mechanism and a disconnect between basic research and clinical application. As a result, finding a solution to overcome this challenge has become an urgent concern in the development of TCM.

In response to the declining rates of drug discovery, mounting difficulties in research and development, and high rates of failure in phase II and III clinical trials, Hopkins introduced the concept of network pharmacology (Hopkins, 2007). For the first time, network pharmacology challenged the traditional approach of developing drugs with high selectivity for a single target and proposed a new model of drug action on biological networks, the “drug-multi-target-disease” approach (Kibble et al., 2015). Network pharmacology employs high-throughput omics data analysis, computer simulations, and network database searches to study the multi-target networks of diseases caused by the actions of compounds. This holistic and systematic model aligns with the comprehensive perspective of TCM, dialectical therapy, and prescription drugs. In recent years, numerous studies using network pharmacology have been conducted to examine the mechanisms of action of traditional Chinese medicine and to discover pharmacodynamic substances and active compounds (Guo et al., 2019; Liu et al., 2021).

Kang-ai Injection (KAI) is a traditional Chinese medicine that combines the properties of Astragalus, Ginseng, and sophora flavescens. It is famous for its beneficial effects, such as enhancing immune function, reducing side effects of chemotherapy, and improving chemotherapy sensitivity (Zhou et al., 2022). It is used to treat various types of cancer, including hepatocellular carcinoma, lung cancer, rectal cancer, and malignant lymphoma (Li et al., 2019; Sun et al., 2021; Zheng et al., 2022). Previous research on KAI had focused on its pharmacodynamic components, the pharmacological effects of its herbs, and clinical studies on its combination with chemotherapy drugs. However, the drug contains many active ingredients that can exert pharmacological effects through multiple targets and pathways. KAI’s active components and exact molecular mechanism in treating intrahepatic cholangiocarcinoma remain largely enigmatic, necessitating further examination.

**FIGURE 1:**
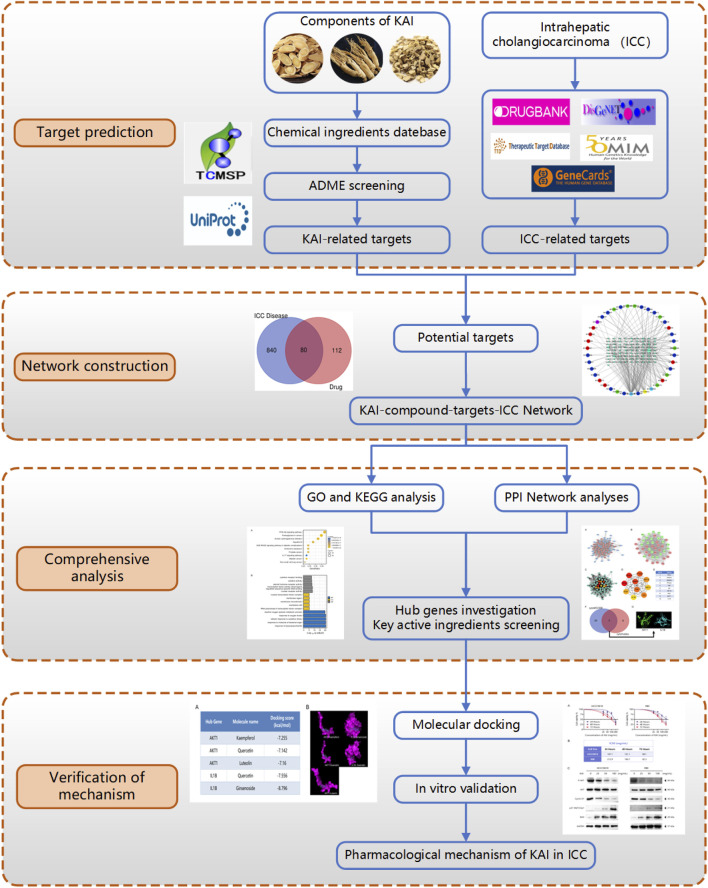
Flow diagram of the research on the mechanism of Kang-ai injection in intrahepatic cholangiocarcinoma.

This research utilized computer simulation, data analysis, and multiple database searches to predict KAI’s primary active components and therapeutic targets. The predictions were then confirmed through molecular docking and *in vitro* cellular research (Figure 1). Concurrently, the target network of drug and disease interaction was established, which provided evidence for elucidating the mechanism of KAI in treating intrahepatic cholangiocarcinoma.

## Materials and methods

### Active ingredients and target collection of KAI

Chang-bai Shan Pharmaceutical Co., Ltd., supplied KAI (Jilin, China). Initially, we screened the active ingredients of Astragalus membranaceus, Ginseng, and Sophora flavescent in KAI through TCMSP, and set the oral bioavailability (OB) ≥ 30% and drug-like (DL) ≥ 0.18 as the active compound ADME screening conditions (Supplementary Table S1). These target proteins of the active drugs were matched with TCMID and the Drug-Bank database. Then, these target proteins are standardized into human species genes through UniProt database.

### Acquisition of disease targets for intrahepatic cholangiocarcinoma

We screened targets related to intrahepatic cholangiocarcinoma by integrating information from OMIM, the Drug-Bank database, the DisGeNET database, and the GeneCardsSuite database platform.

### Construction of protein-protein interaction network (PPI) and screening of hub gene

The targets of the active ingredients in KAI were mapped to coincide with the disease targets of intrahepatic cholangiocarcinoma to acquire the core target proteins for medication therapy. The STRING database was used to build the shared target-gene-protein interaction network; the protein interaction results were loaded into Cytoscape 3.7.2, and two algorithms were used to screen for critical genes. First, we used the cytoHubba function to perform a topological analysis of the network nodes to screen the top ten potential genes; then, we used the cytoMCODE function and the PPI network to screen the sub-networks and selected genes with scores above the median in the sub-network to intersect with the critical genes in the cytoHubba screen; finally, the Hub gene is screened according to the score (Chin et al., 2014; Ma et al., 2021).

### Enrichment analysis of GO and KEGG pathway

The intersection targets of the KAI and ICC disease target network were analyzed for GO and KEGG pathway enrichment through the application of the biological information annotation database (DAVID). The GO analysis notes are divided into three parts, namely, molecular function (MF), cellular component (CC), and biological process (BP). The results were finally visualized by R software (Yu et al., 2012).

### Molecular docking of active components of KAI with target protein

After searching the Uniprot database for the receptor protein encoded by the selected gene, we downloaded the crystal structure of the receptor protein in the RCSB PDB database and the 3D structure of the active ingredient of KAI in the PubChem database. ChemBio 3D software calculated the minimization energy and output 3D structure. The receptor protein was dehydrated using PyMOL 2.4.0 software, and it was hydrogenated and charge calculated by Autodock software. This receptor protein docking site’s parameters were configured to incorporate the active pocket site for small molecule ligand binding. Ultimately, the receptor protein is docked to the active compound’s small molecule ligand using Auto-dock Vina. Lesser the docking score, the more securely the ligand binds to the protein (Trott and Olson, 2010; Koebel et al., 2016; Nguyen et al., 2020).

### Cell proliferation

This study used HCC9810 (Shanghai Branch Cell Bank, China) and RBE (Tohoku University Cell Resource Center, Japan). Cell lines were incubated in RPMI 1640, with 10% FBS (Gibco) and antibiotics (penicillin 100 U/mL, streptomycin 100 mg/mL), at 37°C in an incubator containing 5% CO_2_ (Song et al., 2021).

Utilizing CCK-8 (Dojindo, Kumamoto, Japan), the inhibitory impact of cell growth was detected. HCC9810 and RBE were cultured at a density of 5,000 cells per well in 96-well plates. They were incubated for 8 h before being treated for 24 h, 48 h, or 72 h, respectively, with or without KAI at the stated doses. The tumor cells were washed twice with PBS, treated with a 1:10 dilution of CCK-8 reagent, and incubated for two hours at 37°C. Each day, the absorbance of the cells at 450 nm was observed (Song et al., 2020).

### Western blot

Cell lysates were collected using RIPA buffer containing 0.1% PMSF (BOSTER Biotechnology; Wuhan, China). SDS-PAGE performed separation on a 10% gradient gel. The separated proteins on the gel were transferred to a PVDF membrane of 0.45 μm (Chen et al., 2018). Refer to Table 1 for detailed antibody information.

**TABLE 1:** Antibodies for Westen blot assays.

Gene	Manufacturer	Dilution ratio
P-AKT	Cell Signaling Technology	1:800
AKT	Cell Signaling Technology	1:1,000
Bax	Cell Signaling Technology	1:1,000
Bcl-2	Cell Signaling Technology	1:1,000
Survivin	Cell Signaling Technology	1:800
GAPDH	Beyotime	1:1,000

P-AKT: Phospho-Akt (Ser473).

### Statistical analysis

Statistical analysis was performed using IBM SPSS 23.0 (SPSS, Chicago, IL) and GraphPad Prism 7 (GraphPad Software Inc., San Diego, CA, United States). Continuous variables were expressed as mean ± SEM; comparisons between groups were made using Student *t*-test or Wilcoxon signed-rank test. The categorical data comparison was performed using the χ2 or Fisher’s exact test. Statistical significance was set at *p* < 0.05. All experimental results were independently replicated three times (Hu et al., 2019).

## Results

### Screening active compounds and ICC-associated genes to identify potential ICC therapy targets

**FIGURE 2:**
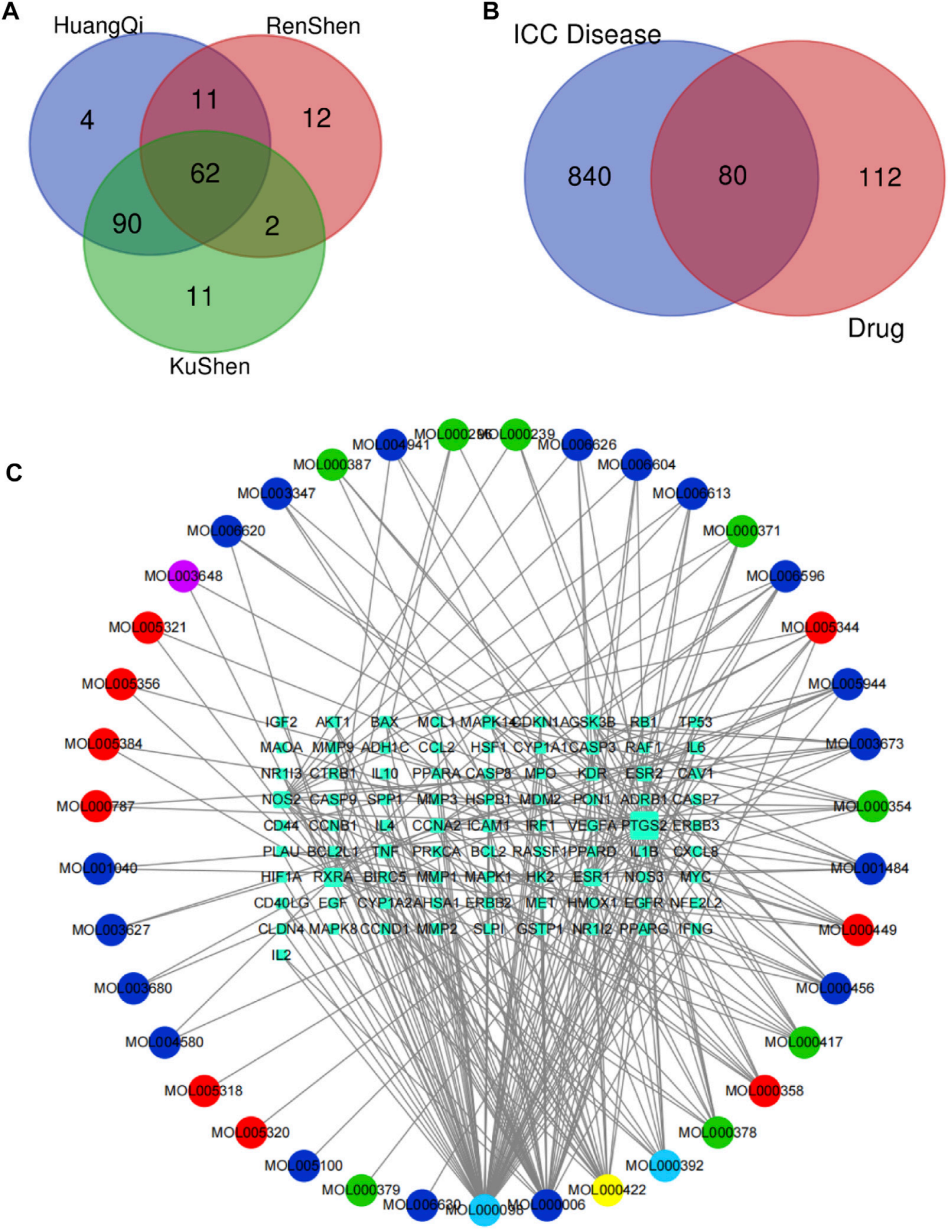
Screening of potential targets and construction of active ingredient-target networks. **(A)** The Vene diagram is composed of the target corresponding to the three main components of Astragalus, Ginseng, and Sophora flavescens. In the Vene diagram, Huangqi corresponds to Astragalus, Renshen corresponds to Ginseng, and Kushen corresponds to Sophora flavescens; **(B)** Identification of the drug-target disease-related genes by taking an intersection of drug-target genes and ICC–related genes; **(C)** The drug-targets interaction pharmacology network. The circle represents the small molecule active compound in KAI. Each color represents a traditional Chinese medicine ingredient, red represents the ingredient from Ginseng, green represents the ingredient from Astragalus membranaceus, blue represents the ingredient from Sophora flavescens, yellow represents the ingredient from Ginseng and Astragalus membranaceus, light blue represents the ingredient from astragalus membranaceus and sophora flavescens, and purple represents the ingredient from ginseng and sophora flavescens; squares represent drug-disease related target genes, node size represents the number of connections to surrounding nodes, and the lines between nodes represent interactions.

By using the TCMSP database, following the active compound ADME screening conditions, we obtained the active compound as a critical component in the drug: Astragalus membranaceus was screened for 20, Ginseng for 22, and Sophora flavescens for 45 active compounds; details are shown in Table 2. 192 drug target genes were obtained from the database (Figure 2A). Moreover, we screened 920 ICC-associated gene collections from the OMIM library, TTD database, DrugBank database, DisGeNET database, and GeneCardsSuite database platform for targets relevant to intrahepatic cholangiocarcinoma. We eventually got the set of potential target genes of ICC for drug therapy by mapping the crossover of drug target genes to disease-related genes (Figure 2B).

**TABLE 2:** The top 10 ingredients in the KAI-ICC network.

Mol ID	Degree	Molecule name
MOL000098	63	quercetin
MOL000006	33	luteolin
MOL000422	19	kaempferol
MOL000392	10	formononetin
MOL000378	9	7-O-methylisomucronulatol
MOL000417	8	Calycosin
MOL000358	8	isorhamnetin
MOL000456	8	beta-sitosterol
MOL000354	7	isorhamnetin
MOL000449	7	Stigmasterol

### Critical gene AKT1 identified for drug therapy through network analysis

**FIGURE 3:**
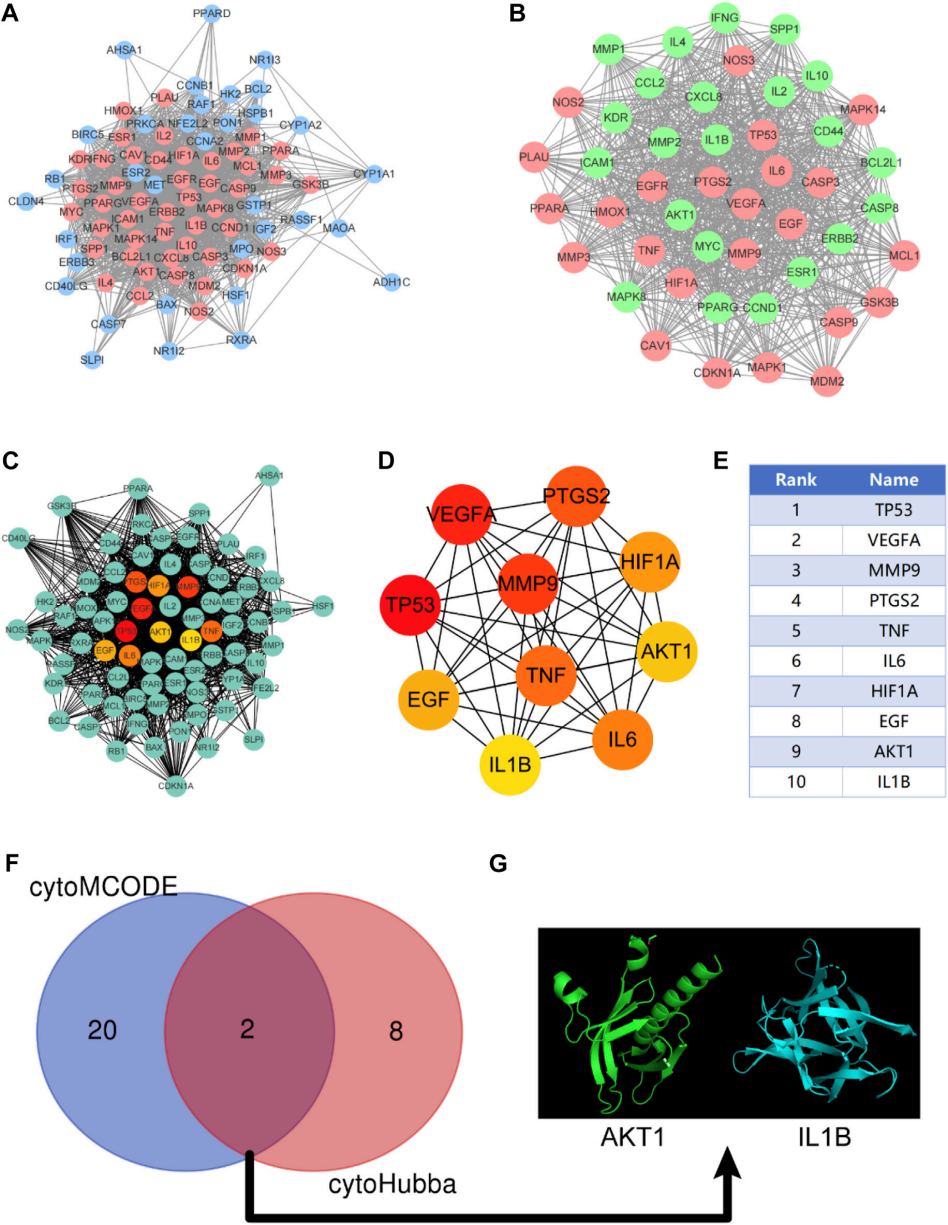
Construction of protein-protein interaction network (PPI) and screening of Hub gene. **(A)** PPI network constructed by KAI-ICC potential target, red represents the sub-network constructed by using the cytoMCODE function; **(B)** The sub-network constructed by cytoMCODE, in which green represents the selected genes in the sub-network with scores higher than the median; **(C–E)** The topology of network nodes is analyzed by using the cytoHubba algorithm to screen the top ten potential genes; **(F, G)** In the sub-network, select the genes with higher than median scores to intersect with the critical genes in the cytoHubba screening to obtain the hub genes.

After discovering the compound-target disease-associated genes, we visualized the drug-active ingredient-target interaction network with 121 nodes and 264 edges using Cytoscape 3.7.2 (Figure 2C). Typically, multiple active compounds focus on a single gene, while a single compound can aim at multiple genes. We considered the degree of the node (Degree value), a crucial indicator to describe the node, to be represented by the number of nodes connected. As the substances with the higher degree values in this network, we discovered that quercetin (MOL000098) assumes the role of crucial compounds (Supplementary Table S1). The topology of network nodes was first analyzed using the cytoHubba function to screen the top ten potential genes (TP53, VEGFA, MMP9, PTGS2, TNF, IL6, HIF1A, EGF, AKT1, IL1β) by PPI protein interaction network analysis. AKT1 was ultimately chosen as our critical gene by the score after the genes were eventually intersected with the cytoMCODE functional screen (Figure 3).

### KEGG and GO Enrichment Analysis Identifies Key Signaling Pathways in Drug Treatment of ICC

**FIGURE 4:**
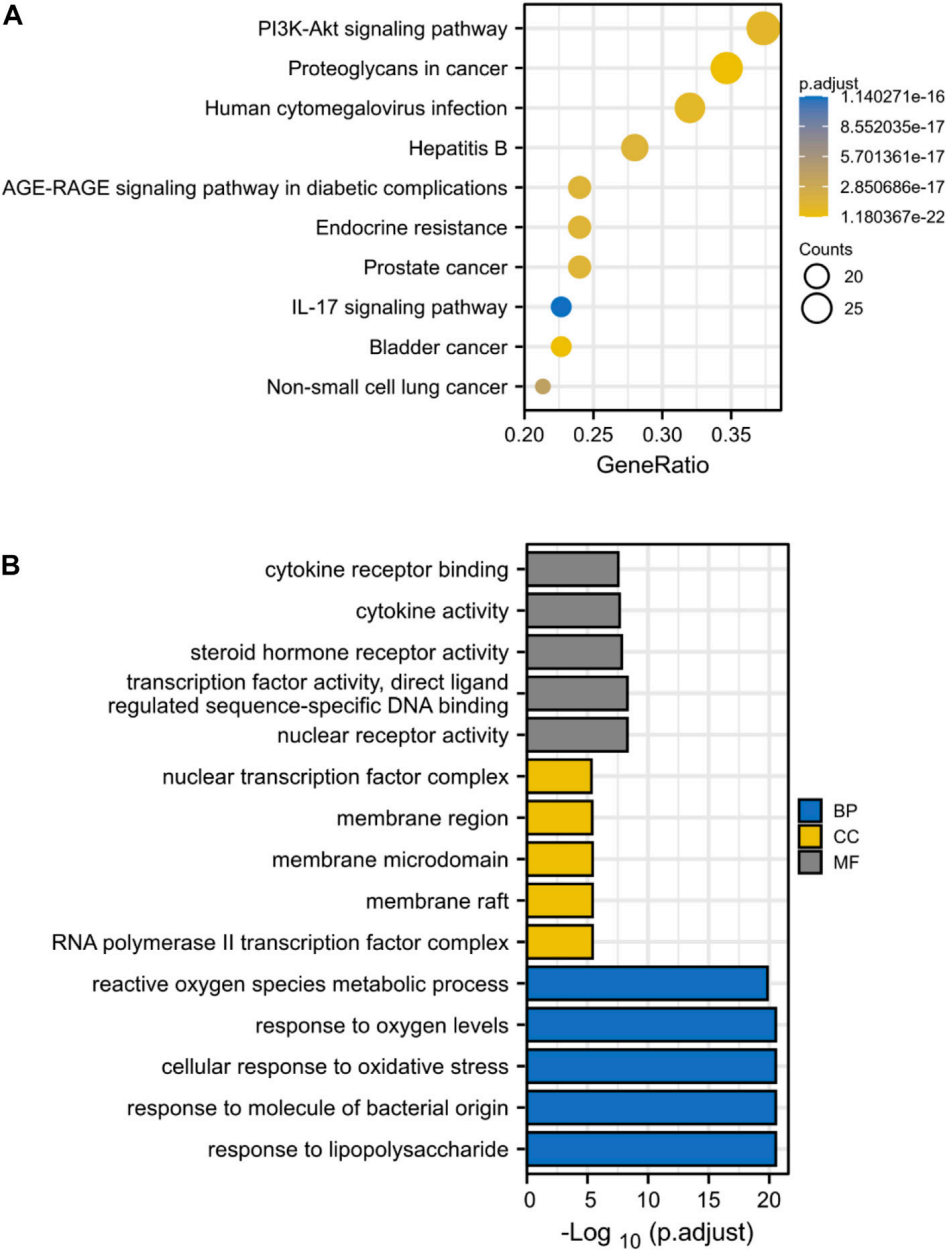
KEGG and GO Enrichment Analysis Identifies Key Signaling Pathways in Drug Treatment of ICC. **(A)** KEGG enrichment analysis, the size of each node indicates enriched counts, the abscissa represents the enriched gene ratio, and color means enriched adjusted *p*-value; **(B)** GO enrichment analysis, blue represents biological process enrichment analysis, yellow represents cell component enrichment analysis, and gray represents molecular function enrichment analysis.

Through KEGG enrichment analysis, 80 target genes enrichment pathways were identified. Figure 4A displays the top ten KEGG signaling pathways with the highest enrichment. We found that PI3K-AKT pathway may play an essential role in the drug treatment of diseases. Potential BP, CC, and MF of 80 target genes were identified by GO enrichment analysis. The top 5 enriched pathways are shown in Figure 4B. The reactive oxygen species metabolic process in BP, the nuclear transcription factor complex in CC, and cytokine receptor binding and activity in MF all serve critical biological functions.

### Validation of hub genes AKT1 and IL1β affinity to active components through molecular docking

**FIGURE 5:**
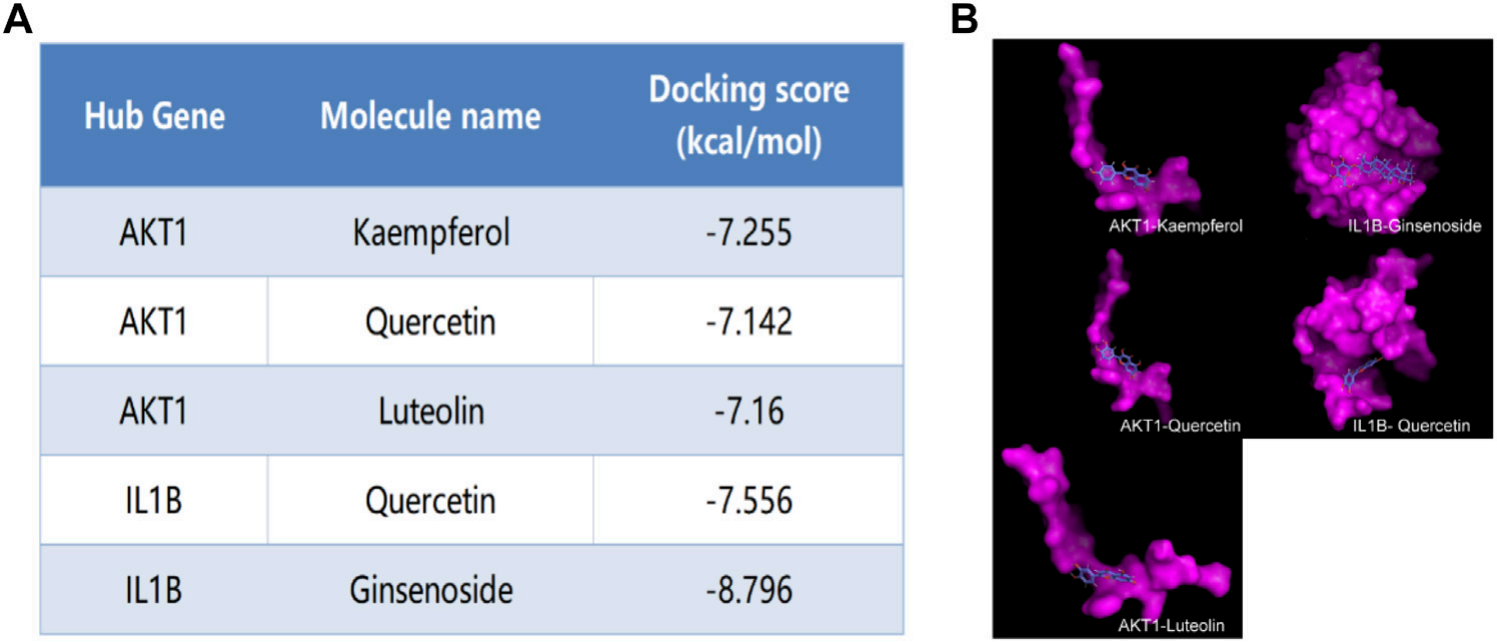
Molecular docking analysis of hub targets and corresponding active compounds. **(A)** Molecular docking score of hub target and corresponding active compound; **(B)** 3D conformational display of molecular docking between hub targets and corresponding active compound.

We selected Hub genes (AKT1, IL1β) for molecular docking validation. Following identifying the docking components based on the target molecules, we got three active compounds against the AKT1 protein and two active compounds against IL1β from the compound-target interaction network for molecular docking. The affinity is judged by binding energy, less than 5.0 kcal/mol, indicating excellent affinity. According to the findings of molecular docking, the binding energies of the core target proteins AKT and IL1β with the active components of KAI are less than 5.0 kcal/mol, respectively (Figure 5A). The outcomes demonstrate that, as shown in Figure 5B, the active ingredients of KAI may easily access and stably bind the active pockets of AKT1 protein and IL1β protein.

### KAI inhibits the proliferation of ICC cell lines by affecting PI3K/AKT pathway

**FIGURE 6:**
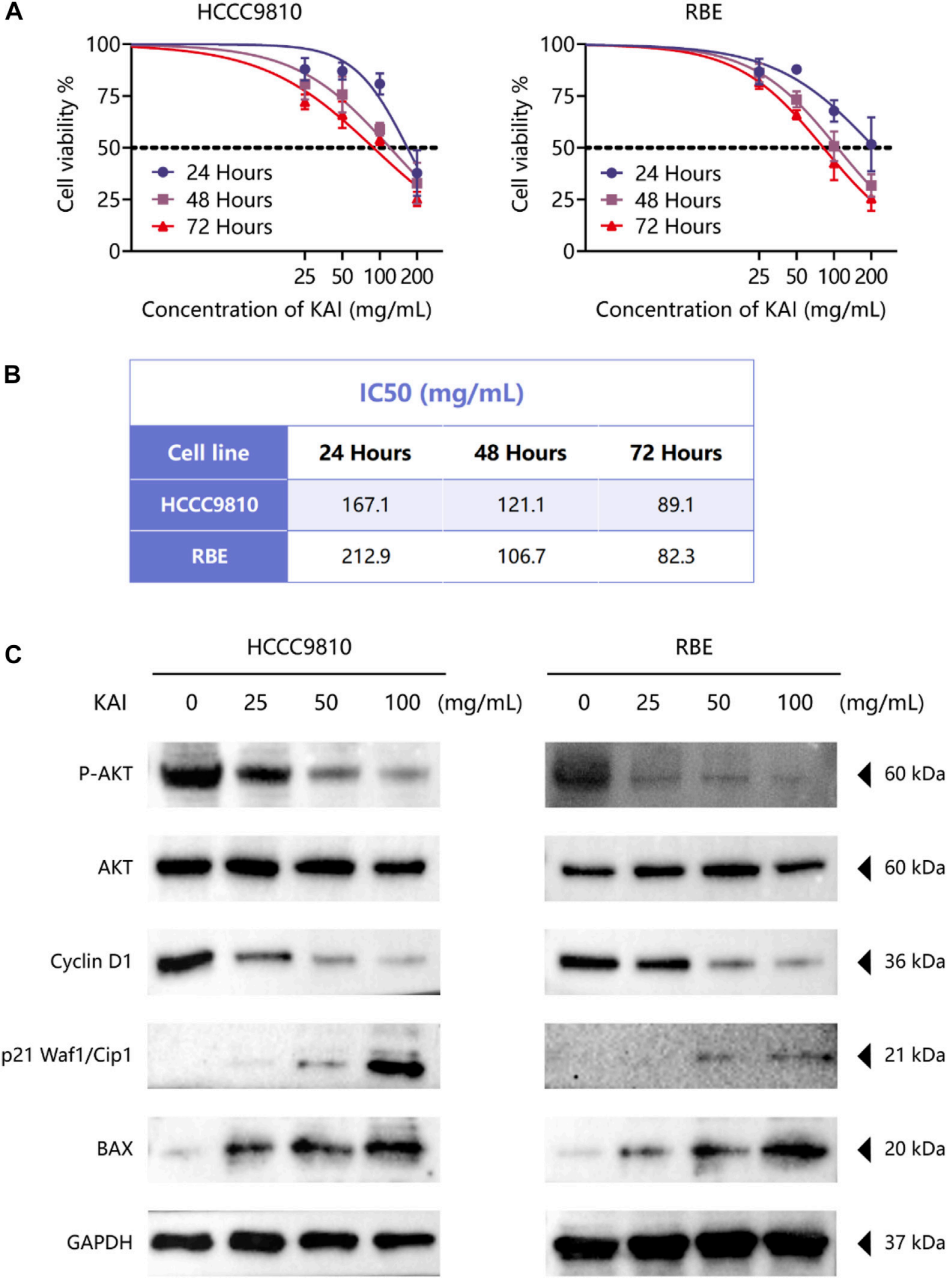
KAI inhibits the proliferation of ICC cell lines by affecting PI3K/AKT pathway. **(A, B)** HCCC9810 and RBE cells were treated with KAI at the indicated concentrations for 24 h, 48 h, or 72 h. Cell viability was measured using CCK8, and IC50 values were calculated; **(C)**. Western blot assay was used to detect the phosphate expression of AKT and the expression of AKT and its downstream proteins in ICC cells in different treatment groups.

The bioinformatics predictions suggest the crucial role of AKT1 as a target and the essential role of the PI3K/AKT Pathway in drug therapy for ICC. Thus, we used CCK-8 assay to detect the proliferation of ICC cell lines treated with different concentrations of KAI and found that it significantly inhibited the growth of tumor cells in a time- and dose-dependent manner (Figure 6A). Figure 6B displays the IC50 concentration values for the ICC cell lines.

**FIGURE 7:**
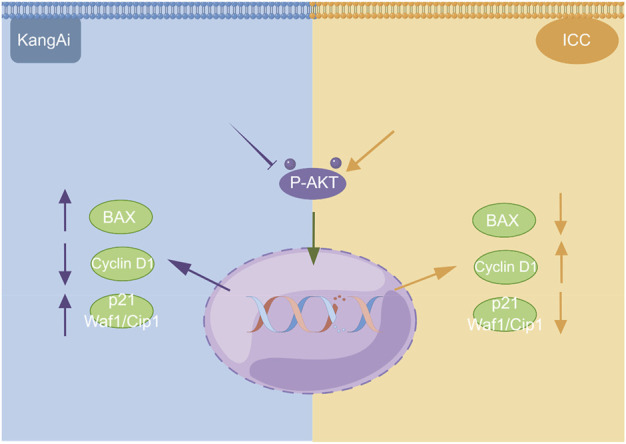
Schematic depiction of the underlying mechanism of KAI inhibiting tumor activity in ICC cells *via* the PI3K/AKT signaling pathway. This image was created with the help of the Fig-draw website, ID: ISRPYd1aa0.

We used western blotting to evaluate the expression levels of PI3K/AKT signaling pathway. The results showed that the expression of P-AKT, p21 Waf1/Cip1, and BAX was significantly reduced, while proteins such as Cyclin D1 were downregulated after treatment of HCCC9810 and RBE with KAI (Figure 6C; Supplementary Figure S1). These findings imply that KAI suppressed ICC cell proliferation by inhibiting PI3K/AKT pathway (Figure 7).

## Discussion

Intrahepatic cholangiocarcinoma is the most common type of primary liver malignancy after hepatocellular carcinoma. Due to its heterogeneity and aggressiveness, effective treatment has been lacking (Dong et al., 2018). TCM has a long history of success, particularly in treating challenging diseases such as major infectious diseases, immune inflammation, and malignant tumors. However, there is often more than one component of TCM, and the role of each component is often complex, which leads to the dilemma of unclear pharmacological mechanisms in the development of TCM. KAI, an injectable solution made using TCM theory, has demonstrated excellent therapeutic effects in clinical studies of several solid tumors, particularly hepatocellular cholangiocarcinoma.

Nevertheless, as the second largest tumor in primary liver cancer, ICC is far more malignant than HCC. There is a lack of research on the efficacy and mechanism of KAI in intrahepatic cholangiocarcinoma. In this research, we utilized network pharmacology and molecular docking to predict the potential targets and mechanisms of the effect of KAI in ICC. Concurrently, the drug’s anti-tumor activity and pharmacological mechanism *in vitro* were verified using HCCC9810 and RBE, two intrahepatic bile duct-derived cell lines.

The main ingredients of KAI are Astragalus membranaceus, Ginseng, and Sophora flavescent, which are isolated from Chinese herbs (Zhang et al., 2018). About 87 active compounds were obtained from our ADME screening of three drugs for active compounds. The combined analysis of the topological isomerization of the drug-compound-target network revealed that the compound quercetin (MOL000098) might play a central role as the drug’s active ingredient. Quercetin, a flavonoid compound, has demonstrated its inhibitory effects on tumor growth and invasion in various tumors (Tsou et al., 2016; Hu et al., 2017; Paller et al., 2018). Li S et al. showed that quercetin could enhance the synergistic effect of chemotherapeutic drugs on breast cancer cells while decreasing their toxic effects (Li et al., 2018). Quercetin also has anti-aging activity, and it can rejuvenate cells by regulating various cellular processes related to cell cycle, chromosome cohesion, and antioxidants (Geng et al., 2019). However, quercetin’s poor water solubility and oral availability limit its application as an anti-tumor agent (Hu et al., 2017). In order to improve the bioavailability and stability of quercetin, Kaili Hu et al. phosphorylated quercetin hydroxyl groups to increase the aqueous solubility of hydrophobic drugs (Hu et al., 2017). KAI, as an injectable aqueous solvent, can very well avoid the poor oral utilization of quercetin. In addition, our molecular docking experiments revealed that quercetin, as the active compound, fitted well with the core target protein identified by the PPI protein interaction network (Figure 2C). Then we speculate that quercetin may be an essential active compound in KAI and play a subsequent biological function by combining with the core target protein.

KAI, one of the traditional injections of Chinese medicine, exhibits excellent anti-tumor activity and synergistic effects of chemotherapeutic drugs in various solid tumors (Cang et al., 2022; Pu et al., 2022). However, the possible mechanism of KAI in cancer therapy remains obscure. Pu Q et al. showed that KAI might inhibit cell death and modulate the cytotoxicity of chemotherapeutic agents in lung adenocarcinoma through cellular autophagy (Zhou et al., 2022). Our network pharmacology predictions and *in vitro* experiments both support the significant role played by the PI3K-AKT pathway in the mechanism of drug action in ICC (Figure 6). PI3K/AKT signal pathway is one of the primary growth regulation pathways in normal cells or cancer, in which AKT plays a vital role as the central node of the signal pathway (Kim et al., 2017; Zhang et al., 2017). PI3K/AKT signaling pathway regulates cell proliferation, cell cycle progression, and apoptosis through the phosphorylation of protein kinase B (also known as AKT) (Le et al., 2016). Western blot analysis showed that the expression of P-AKT1 in drug-treated ICC cells was reduced. Combining the molecular docking results of AKT1, we believe that AKT1, as a core target protein, changes its phosphorylation activity by binding with multiple active compounds of KAI.

In addition, our research also found that IL1β, another critical target molecule screened out, may also exert a potential role. As a cellular inflammatory factor secreted by various immune cells, such as macrophages, IL1β is often released together with proinflammatory factors after hepatocyte apoptosis, such as IL-6 and TNF-α (Guo et al., 2020). We speculate that KAI may affect the tumor immune microenvironment by releasing immune factors, which may provide synergy for future immune therapy. Among the genes screened using the cytoMCODE algorithm, we believe that TP53, VEGFA, MMP9, and HIF-1A, which rank first in the score, also play an essential role. TP53 encodes tumor suppressor p53, the most common mutated gene in human cancer (Boettcher et al., 2019). TP53, as a critical protein involved in multiple signal pathways, including Wnt and Akt, regulates cell proliferation and apoptosis (Chaudhary et al., 2018). Matrix metalloproteinase 9 (MMP9) is involved in the tumor cells’ metastatic invasion as a downstream EMT transcription factor (Gujral et al., 2014; Aiello et al., 2018). Hypoxia is a significant driver of cancer invasion and metastasis, and HIF-1A and HIF-2A, as central regulators of the cellular response to hypoxia, play an essential role in this process (Liu et al., 2015; Vukovic et al., 2016). Overall, the pharmacological network of interaction between active components and targets reflects the characteristics of multi-components, multi-targets, and multi-pathways, which is consistent with the overall concept of TCM.

This study also has several limitations. Firstly, our study lacks further validation of the efficacy and safety of the drugs *in vivo*; secondly, this study lacks further verification of these phenotypes of immune infiltration, which may be of value for future anti-tumor collaboration with immunotherapy. Further data should be collected in our subsequent preclinical studies to verify anti-tumor activity, toxic effects, and adverse events.

In conclusion, our study demonstrated for the first time the anti-tumor efficacy of KAI in ICC, and its key components, target proteins, and signaling pathways were predicted using network pharmacology, molecular docking, and *in vitro* validation. It also revealed that the anti-tumor effect of KAI is mainly *via* inhibition of AKT phosphorylation levels, thereby inhibiting PI3K/AKT signal, leading to changes in tumor cell proliferation of tumor cells.

## Data Availability

The datasets presented in this study can be found in online repositories. The names of the repository/repositories and accession number(s) can be found in the article/Supplementary Material.

## Supplementary material

Supplementary Figure S1 A Densitometry analysis was performed on three experiments representative of Figure 6C and expressed relative to GAPDH or the corresponding total protein as the internal control. All bar graphs depict the quantification of triplicate results with mean ± SD values. *P < 0.05; **P < 0.01; ***P < 0.001; ****P < 0.0001. (Image J 1.46r software; National Institutes of Health, Bethesda, MD).

Supplementary Table S1 Active components of KAI.

## Abbreviation

ICC, intrahepatic cholangiocarcinoma; KAI, Kang-ai injection; CCK8, Cell Counting Kit-8; FBS, fetal bovine serum; IL1β, Interleukin-1 beta; BAX, Apoptosis regulator BAX; Cyclin D1, Cyclin-D1-binding protein 1; KEGG, Kyoto Encyclopedia of Genes and Genomes; MMP-9, matrix metallopeptidase 9; AKT1, RAC-alpha serine/threonine-protein kinase; GAPDH, glyceraldehyde 3-phosphate dehydrogenase; HIF1A, Hypoxia-inducible factor 1-alpha; HIF-2A, Hypoxia-inducible factor 2-alpha; TCMSP, Traditional Chinese Medicine Systems Pharmacology Database and Analysis Platform (http://lsp.nwu.edu.cn/tcmsp.php); TCMID, Traditional Chinese Medicines Integrated Database (http://www.megabionet.org/tcmid/); The Drug-Bank database, (https://www.drugbank.ca/); UniProt database, (http://www.uniprot.org/uploadlists/); OMIM, Online Mendelian Inheritance in Man (http://www.omim.org/); DisGeNET database, (https://www.disgenet.org/); GeneCardsSuite database platform, (https://www.genecards.org/); STRING database, (https://string-db.org/); RCSB PDB database, (http://www.rcsb.org/pdb/home/home.do); PubChem database, (https://pubchem.ncbi.nlm.nih.gov/).
