# Supplementary material for: Uncovering the mechanism of Kang-ai injection for treating intrahepatic cholangiocarcinoma based on network pharmacology, molecular docking, and in vitro validation
Source: Front Pharmacol. 2023 Mar 2;14:1129709. doi: 10.3389/fphar.2023.1129709 (PMC10017963; doi:10.3389/fphar.2023.1129709)
Supplement: Supplementary file 2 [file DataSheet1.docx]

### Supplementary materials Figure S1


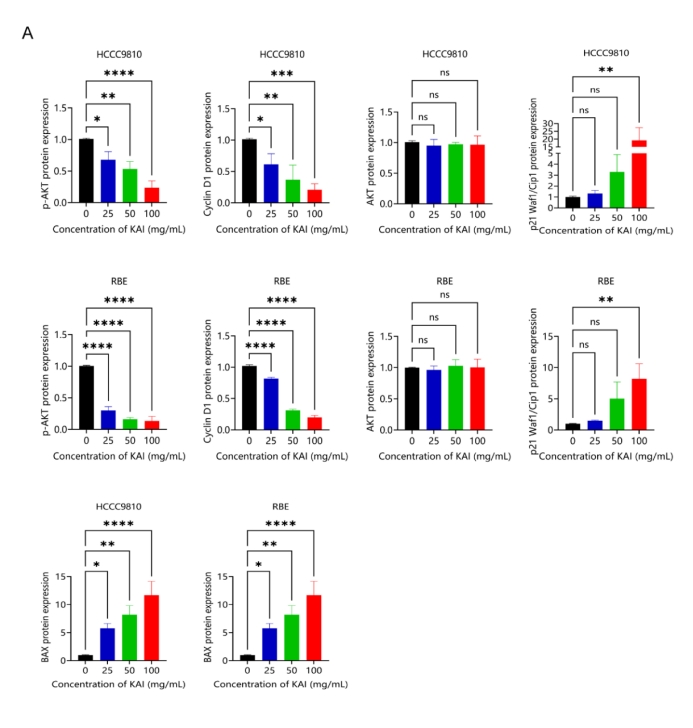


1. Densitometry analysis was performed on three experiments representative of Figure 6C and expressed relative to GAPDH or the corresponding total protein as the internal control. All bar graphs depict the quantification of triplicate results with mean ± SD values. *, P < 0.05; **, P < 0.01; ***, P < 0.001; ****, P < 0.0001. (Image J 1.46r software; National Institutes of Health, Bethesda, MD)
